# Multiple Selection Signatures in Farmed Atlantic Salmon Adapted to Different Environments Across Hemispheres

**DOI:** 10.3389/fgene.2019.00901

**Published:** 2019-10-01

**Authors:** María Eugenia López, Tyler Linderoth, Ashie Norris, Jean Paul Lhorente, Roberto Neira, José Manuel Yáñez

**Affiliations:** ^1^Facultad de Ciencias Veterinarias y Pecuarias, Universidad de Chile, Santiago, Chile; ^2^Department of Animal Breeding and Genetics, Swedish University of Agricultural Sciences, Uppsala, Sweden; ^3^Department of Integrative Biology, University of California, Berkeley, CA, United States; ^4^Marine Harvest, Kindrum, Fanad, C. Donegal, Ireland; ^5^Benchmark Genetics Chile, Puerto Montt, Chile; ^6^Facultad de Ciencias Agronómicas, Universidad de Chile, Santiago, Chile; ^7^Núcleo Milenio INVASAL, Concepción, Chile

**Keywords:** selection signatures, *Salmo salar*, Domestication, SNP data, artificial selection

## Abstract

Domestication of Atlantic salmon started approximately 40 years ago, using artificial selection through genetic improvement programs. Selection is likely to have imposed distinctive signatures on the salmon genome, which are often characterized by high genetic differentiation across population and/or reduction in genetic diversity in regions associated to traits under selection. The identification of such selection signatures may give insights into the candidate genomic regions of biological and commercial interest. Here, we used three complementary statistics to detect selection signatures, two haplotype-based (iHS and XP-EHH), and one F_ST_-based method (BayeScan) among four populations of Atlantic salmon with a common genetic origin. Several regions were identified for these techniques that harbored genes, such as *kind1* and *chp2*, which have been associated with growth-related traits or the *kcnb2* gene related to immune system in Atlantic salmon, making them particularly relevant in the context of aquaculture. Our results provide candidate genes to inform the evolutionary and biological mechanisms controlling complex selected traits in Atlantic salmon.

## Background

Domestication is a complex evolutionary process whereby wild animals or plant populations adapt to environmental conditions created by humans and so involves genetic and developmental changes over multiple generations (Price, 1984; Liu et al., 2017). Since the beginning of domestication, humans have exploited the genetic diversity of various species to model them according to their needs (Driscoll et al., 2009). This has been amplified since the establishment of explicit genetic improvement objectives. As a result of intense selection pressure, dramatic phenotypic changes (Rubin et al., 2012) and substantial and continued genetic improvement have been made in domestic populations over the past decades (Hill and Bunger, 2004).

Domestication in most fish is relatively recent compared with terrestrial animals (Teletchea and Fontaine, 2014; López et al., 2015), but has expanded rapidly over the last decades (Lorenzen et al., 2012), and several breeding programs have been implemented in different aquatic species, such as tilapia (*Oreochromis niloticus* L), rainbow trout (*Oncorhynchus mykiss* W), coho salmon (*Oncorhynchus kisutch* W), and Atlantic salmon (*Salmo salar* L) among others (Gjedrem, 2010; Gjedrem, 2012; Yáñez et al., 2014). The latter has become one of the most important aquaculture species (FAO, 2016), since it was first farmed in Norway during the 1960s. Despite a generation interval of 3 to 4 years, breeding programs have achieved rapid improvement of economically important traits, such as growth, sexual maturation, and disease resistance (Gjedrem et al., 2012). Domestication and subsequent artificial selection have produced stark phenotypic changes in farmed Atlantic salmon populations (Glover et al., 2017), as evidenced by differences in traits, such as growth and predator awareness, between wild and farmed populations (Thodesen et al., 1999; Glover et al., 2009; Solberg et al., 2012) (Einum and Fleming, 1997).

Positive selection pressures (natural and artificial) experienced by population undergoing selection will cause the frequency of alleles underlying favorable traits to increase rapidly. Linkage disequilibrium (LD) between favorable mutations and neighboring loci will increase and spread, given that there is little opportunity for recombination over the brief time since the onset of intense selection (Sabeti et al., 2002). Analyses of these selection signatures in domestic animals can provide further insights into the genetic basis of adaptation to diverse environments and genotype/phenotype relationships (Oleksyk et al., 2010; Andersson, 2012). Access to genomic data through next-generation sequencing and high-throughput genotyping technologies have made the comparison of genomic patterns of single nucleotide polymorphism (SNP) variation between different livestock breeds possible, allowing for the identification of putative genomic regions and genes under selection in several terrestrial domestic species, including cattle (e.g., Taye et al., 2017), horses (e.g., Avila et al., 2018), sheep (e.g., Ruiz-Larrañaga et al., 2018), and pigs (e.g., Gurgul et al., 2018).

There are several approaches for detecting genomic selection signatures, one of which relies on the length or variability of haplotypes. Directional selection acting on a new, beneficial mutation causes the haplotype harboring the mutation to increase in frequency and to be longer than average. To exploit this pattern for detecting positive selection, Sabeti et al. (2002) proposed the extended haplotype homozygosity (EHH) statistic, which is specifically the probability that two randomly selected haplotypes are identical-by-descent over their entire length around a core SNP (Sabeti et al., 2002). This concept forms the basis for other haplotype homozygosity-based metrics, such as the relative EHH (REHH) (Sabeti et al., 2002) and the widely used integrated haplotype score (iHS) (Voight et al., 2006). iHS compares EHH between derived and ancestral alleles within a population and has the most power to detect selection when the selected allele is at intermediate frequencies in the population (Sabeti et al., 2006; Voight et al., 2006). To detect selection signatures between populations, the cross-population extended haplotype homozygosity test (XP-EHH) compares the integrated EHH profiles between the two populations in the same SNP. This test was designed to detect ongoing or nearly complete selective sweeps in one population (Sabeti et al., 2007). An alternative approach for identifying selection signatures when there are multiple populations for comparison is divergence-based methods, which focus on identifying outlier loci with either higher or lower allele frequency differences among populations than expected without selection (Beaumont and Balding, 2004; Foll and Gaggiotti, 2008; Excoffier et al., 2009). One common approach for quantifying the degree of genetic differentiation between populations is through the fixation index, F_ST_, (Wright, 1951). An unusually high F_ST_ value at a given locus can be indicative of directional selection. Divergence approaches to identify signals of selection have been successful in several domestic species including swine (Cesconeto et al., 2017), sheep (Manunza et al., 2016), and cattle (Maiorano et al., 2018) among others.

Although previous studies have already been carried out to detect selection signatures in Atlantic salmon (Mäkinen et al., 2014; Gutierrez et al., 2016; Liu et al., 2017; López et al., 2018), using multiple different strains adapted to different culture conditions across hemispheres, to explore how genetic variation among them differs, has not been done yet. Herein, we used an Affymetrix 200K SNP array data set to investigate selection signatures in farmed Atlantic salmon populations from the same origin, and subsequently cultivated in Ireland and Chile. We found evidence of selection using two haplotype-based approaches iHS and XP-EHH and one F_ST_-based method, BayeScan, in the genomes of four Atlantic salmon populations. These findings are important because they highlight regions of the genome that might benefit economically relevant attributes, such as growth, resistance to local diseases, and adaptation to specific environmental conditions.

## Materials and Methods

### Samples, Genotyping, and Quality Control

This study was performed using a total of 270 individuals from four populations (Pop-A, n = 40; Pop-B, n = 71; Pop-C, n = 85; Pop-D, n = 74) derived from the Mowi strain. This strain comes from one of the first farmed Atlantic salmon populations, which was established with fish from west coast rivers in Norway, with major contributions from River Bolstad in the Vosso watercourse, River Årøy, and possibly from the Maurangerfjord area (Verspoor et al., 2007). Salmon from the Vosso and Årøy rivers are characterized by large size and late maturity (Verspoor et al., 2007). Phenotypic selection for growth, late maturation and fillet quality was the focus in this population until 1999 (Glover et al., 2009). Ova from this population were imported into the Fanad Peninsula, Ireland, between 1982 and 1986 to establish an Irish-farmed population (Norris et al., 1999). Individuals from this population comprise Pop-A, which we estimate had been under artificial selection for growth for at least 10 generations prior to sampling. Similarly, ova from this farmed, Irish population were introduced into Chile in the early 1990s to establish separate farmed populations in the Los Lagos Region (42°S 72°O) and the Magallanes Region (53°S 70°O). Pop-B and Pop-C correspond to samples from two different populations in the Los Lagos Region that were initially founded with fish from different year-classes. Samples from Pop-D represent one population founded in the Magallanes Region. The three Chilean populations were subsequently adapted to the biotic and abiotic conditions present in southern hemisphere. These populations experienced four generations of selective breeding for growth in Chilean farming conditions prior to sampling, which occurred at the same time that Pop-A was sampled in 2014.

All populations were genotyped using Affymetrix’s Atlantic salmon 200K SNP Chip described in Yáñez et al. (2016). We performed SNP quality control using the Axiom Genotyping Console (GTC, Affymetrix) and SNPolisher (an R package developed by Affymetrix), which i) removed SNPs that did not conform high-quality clustering patterns as outlined by Affymetrix, ii) removed SNPs with genotype call rate lower than 95%, and iii) discarded individuals with genotyping call rate under 90%. As part of the validation of the SNPs chip used in this study, Yáñez et al. (2016) identified loci significantly deviating from Hardy–Weinberg equilibrium in eight populations separately and removed these sites if they were deviating from Hardy–Weinberg equilibrium among all populations. In addition, we limited our analyses to SNPs that mapped to chromosomes in the newest version of the Atlantic salmon reference genome, ICSAG_v2 (GenBank: GCA_000233375.4), which comprised 149,060 SNPs.

### Genetic Diversity, LD, and Population Structure

We evaluated genetic diversity in terms of the observed heterozygosity (H_O_) and expected heterozygosity (H_E_) calculated with PLINK v1.09 (Purcell et al., 2007). We calculated the pair-wise LD as the Pearson’s squared correlation coefficient (*r*^2^) for each population and within chromosomes using PLINK v1.09 (Purcell et al., 2007). For each SNP pair, bins of 100 kb were created based pairwise distance. To investigate population structure, we performed a principal component analysis (PCA) based on genotypes as implemented in PLINK v1.09 and inferred individual ancestry proportions with ADMIXTURE 1.2.2 (Alexander et al., 2009). For the admixture analysis, we performed 200 bootstraps with a number of ancestral lineages (K) ranching from 1 to 20. Ten-fold cross validation (CV = 10) was specified, and we retained results from the K having the lowest cross-validation error. The aforementioned analyses were conducted using a total of 21,950 SNPs, which had a minor allele frequency (MAF) larger than 0.05, were in Hardy–Weinberg equilibrium, and which had LD values of at most 0.4 (to minimize possible confounding effects of LD on the patterns of genetic structure).

### Selection Signatures, Gene Annotation, and Functional Analyses

To identify genomic regions harboring selection signatures, we used one within population iHS and two between-population methods (XP-EHH and BayeScan) over a subset of 120,316 SNPs that had MAF > 0.05 among all populations.

**(1) iHS**. The iHS score for detecting selection is based on the ratio of EHH for haplotypes anchored with the ancestral versus derived allele. The ancestral allele state for our Atlantic salmon populations is unknown and so to avoid losing SNPs by trying to polarize them from publicly available outgroup references, we assumed that the major allele represented the ancestral state as in Bahbahani et al. (2015). We phased the haplotypes using Beagle v.5.0 (Browning and Browning, 2009). Single-site iHS values across the genome were calculated for each populations using the REHH package (Gautier and Vitalis, 2012). These per site iHS values were standardized so that they were approximately distributed according to a standard normal distribution. We required candidate-selected regions to have at least two SNPs ≤ 500 kb apart, each with iHS scores with -log_10_(*p* value) of at least three (*p* value ≤ 0.001) based on a one-tailed test assuming that the standardized iHS ∼ N(0,1).

**(2) XP-EHH**. The XP-EHH statistic compares the integrated EHH between two populations at the same SNP, to identify selection based on overrepresented haplotypes in one of the populations (Sabeti et al., 2007). We evaluated three different pairs of populations with this method Pop-B/Pop-A, Pop-C/Pop-A, and Pop-D/Pop-A. This design was used because of the main objective of this study was to assess how selective pressures have affected populations cultivated in Chile, relative to their founding population, Pop-A, which was used as the reference population. Therefore, we excluded the comparisons between Chilean populations. The XP-EHH statistics were calculated as ln(I_PopO_/I_PopR_), where I_PopO_ is the integrated EHH for the observed populations and I_PopR_ is the integrated EHH value of the reference population. Negative XP-EHH scores suggest selection in the “reference” population, whereas positive scores suggest selection acting in the “observed” population. A -log_10_(*p* value) of three (*p* value ≤ 0.001) was used as the lower threshold for considering XP-EHH score as significant evidence of selection and at least two SNPs ≤ 500 kb apart.

**(3) BayeScan**. We used the Bayesian likelihood method implemented in BayeSCAN v.2.1 to estimate the posterior probability that loci are experiencing selection (Foll and Gaggiotti 2008). This method models allele frequencies in subpopulations derived from a single ancestral population using Dirichlet distributions, which allows for estimating the degree of coancestry within each of these subpopulations through the sum of population-specific, β, and locus-specific, α, effects, making outlier detection robust to confounding complex demographic histories. By estimating the posterior probabilities for both the model including both effects and the model omitting the locus-specific effect, the posterior probability (and posterior odds) for selection at a specific locus can be obtained. When α > 0 for a specific locus, it is evidence of directional selection acting on that locus, whereas α < 0 suggests balancing or purifying selection. This method was run with 5,000 burn-in iterations, followed by 10,000 iterations with a thinning interval of 10. We evaluated the same three pairs of populations of XP-EHH method: Pop-B/Pop-A, Pop-C/Pop-A, and Pop-D/Pop-A. We considered candidate loci under selection as those having a Bayes factor of at least 32 (-log_10_ = 1.5) and a positive value of α (directional selection), corresponding to a posterior probability of 0.97 and considered as being “very strong” evidence of selection and as in iHS and XP-EHH, we required the candidate selected regions to have at least two SNPs ≤500 kb apart.

### Gene Functional Annotation

Genomic regions harboring SNPs showing evidence of selection were annotated based on the ICSAG_v2 reference genome (Lien et al., 2016). We defined the position of the first and last SNP as boundaries of regions putatively under selection using BedTools (Quinlan and Hall, 2010). Gene transcripts from these candidate regions were aligned (using blastx) (Altschul et al., 1990) to the zebra fish (*Danio rerio*) peptide reference database (downloaded from http://www.ensembl.org/) to determine gene identify. As evidence of homology, we used an e-value ≃ 0 and then retrieved the zebra fish gene identifiers information from the ensemble biomart database (http://www.ensembl.org/index.html). Functional annotation of detected genes was performed using DAVID (Huang et al., 2009) with gene list of zebra fish (*Danio rerio*) as reference in Gene Ontology (GO) analysis.

## Results

### Genetic Diversity and Structure

**Figure 1:**
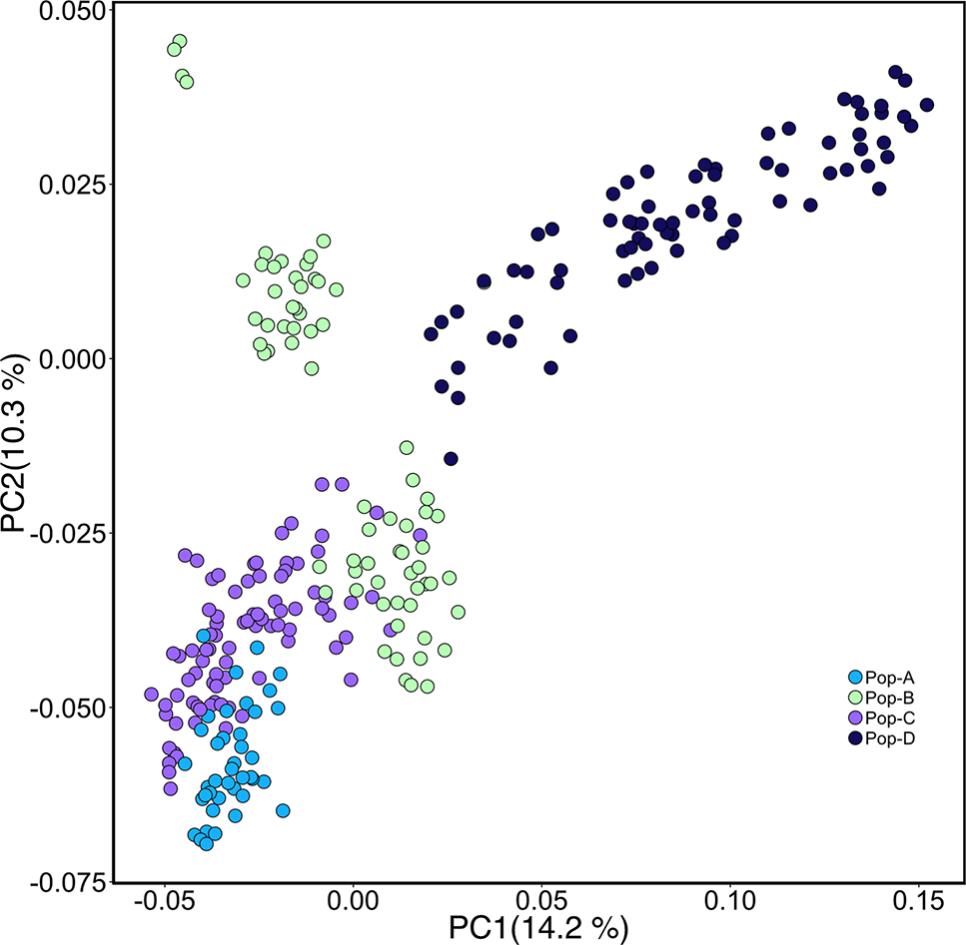
Principal components analysis (PCA) of genetic differentiation among individuals. Each point represents one individual, and different colors represent populations.

**Figure 2:**
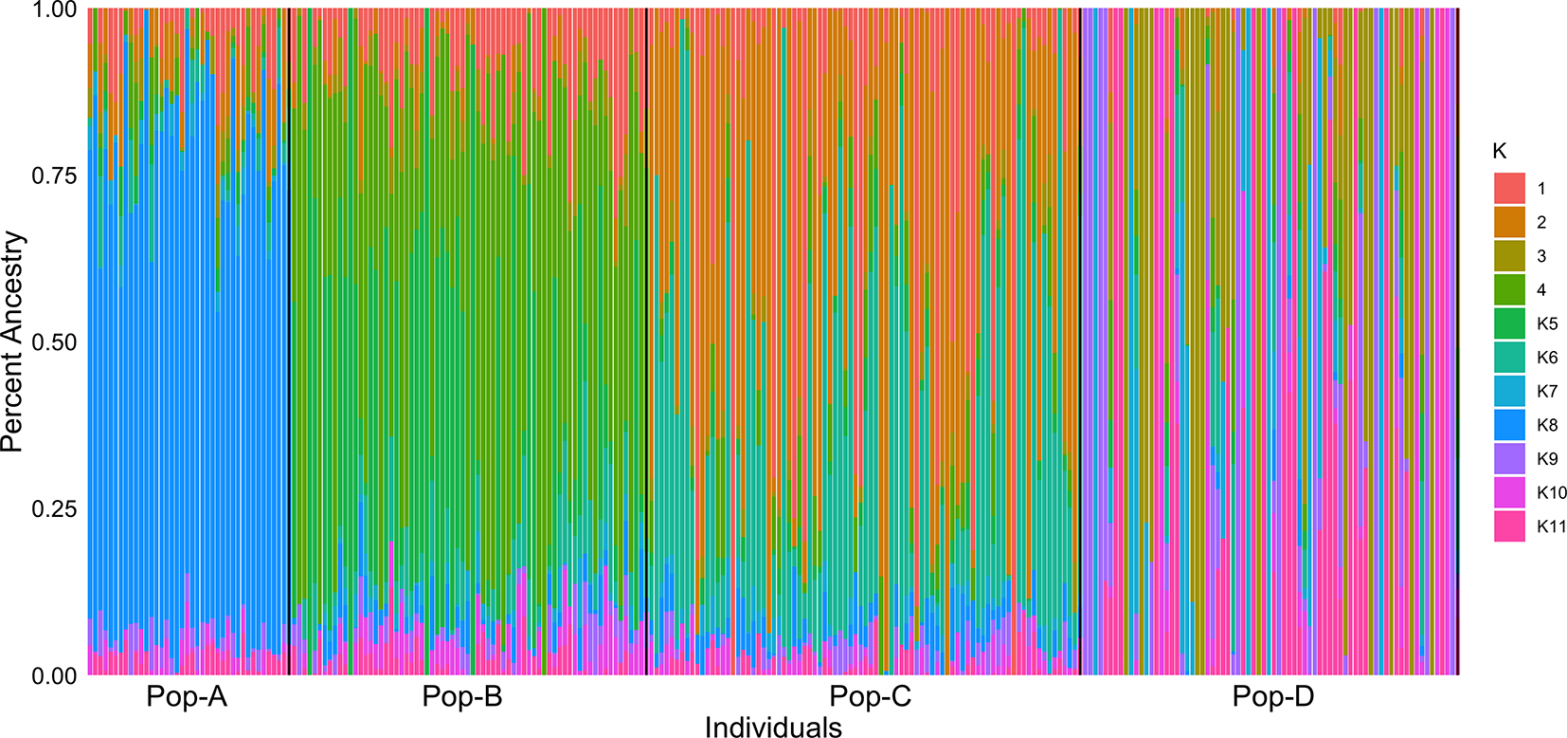
Individual assignment probabilities generated with ADMIXTURE (1⩽K⩽11). Each color represents a cluster, and the ratio of vertical lines is proportional to assignment probability of and individual to each cluster.

We performed PCA based on genotypes to look at the genetic relationship among individuals in our sample. The first and second components accounted for 14.2% and 10.3% of the genetic variation, respectively (**Figure 1**). Pop-A and Pop-C showed close genetic relationship to each other and were most distant to Pop-D from the Magallanes Region along PC1. Pop-B lies between the Pop-A/Pop-C cluster and Pop-D along PC1, with some overlap with Pop-C, which was introduced into the same Los Lagos Region as Pop-B. Overall, principal components showed low genetic variation between populations, but higher within populations, especially in Pop-D that exhibits the most difference among individuals along PC1. Also noteworthy is that Pop-D, with the highest observed heterozygosity (**Table 1**), is uniformly farther to the other farmed populations, except for some individuals from Pop-B. We also performed an Admixture analysis to determine the composition of ancestral lineages among individuals. We found that 11 ancestral lineages were optimal for describing the ancestry of the individuals across the four populations (**Figure 2**). Consistent with the PCA and having the lowest heterozygosity, Pop-A individuals are all relatively the most similar among the populations in terms of their ancestral proportions, being dominated by one ancestral lineage. In contrast, Pop-D individuals tend to be dominated by a single ancestral lineage, but among individuals, the represented lineages are quite different, which is consistent with Pop-D individuals being quite different from each other in the PCA. Pop-B and Pop-C show similar degrees of mixed ancestry, though the dominant lineage is different between the two.

**Figure 3:**
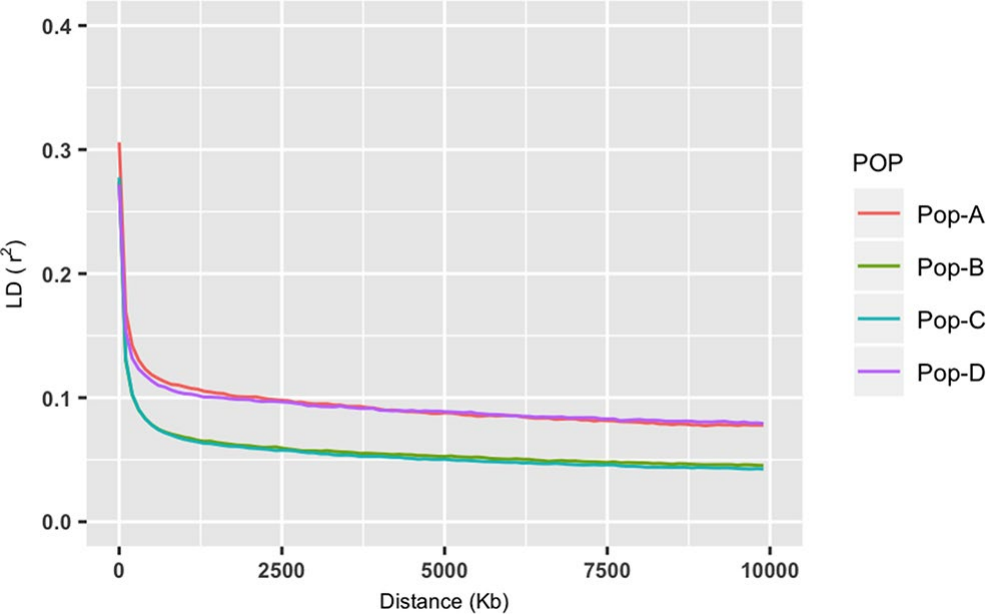
Decay of average linkage disequilibrium (r^2^) over distance across the four farmed populations. Different color lines represent populations: Pop-A = Red, Pop-B = Green; Pop-C = Turquoise and Pop-D = Purple.

Observed heterozygosity levels were similar across the four domestic populations and were slightly higher than expected for populations A, B, and C, and even more so for population D. All these genetic diversity measures were statistically significant (*p* < 0.05, Kruskal–Wallis test) (see **Table 1**). Overall LD results revealed similar patterns for Pop-A and Pop-D, which presented longer range of LD and slower decay in comparison with Pop-B and Pop-C, that also presented similarity between them and a substantial faster LD decay (**Figure 3**). LD measures (*r*^2^) of each chromosome and population are shown in **Table S1** and **Figure S1**. Similar patterns were observed when the chromosomes were analyzed separately. Nevertheless, LD decay in Pop-A was noticeably stronger in chromosomes 2, 9, 19, and 29, whereas LD decay in Pop-D was stronger in chromosomes 13, 17, and 26 (**Figure S1**).

**Table 1:** Genetic diversity values in terms of Observed heterozygosity (H_o_) and Expected heterozygosity (H_E_) across four Atlantic salmon populations used in this study.

Population	H_o_	H_e_
Pop-A	0.4 ± 0.13	0.39 ± 0.11
Pop-B	0.41 ± 0.11	0.41 ± 0.1
Pop-C	0.41 ± 0.11	0.41 ± 0.1
Pop-D	0.47 ± 0.17	0.39 ± 0.11

All these genetic diversity measures were statistically significant (p < 0.05, Kruskal–Wallis test).

### Candidate Regions Under Selection—iHS

**Figure 4:**
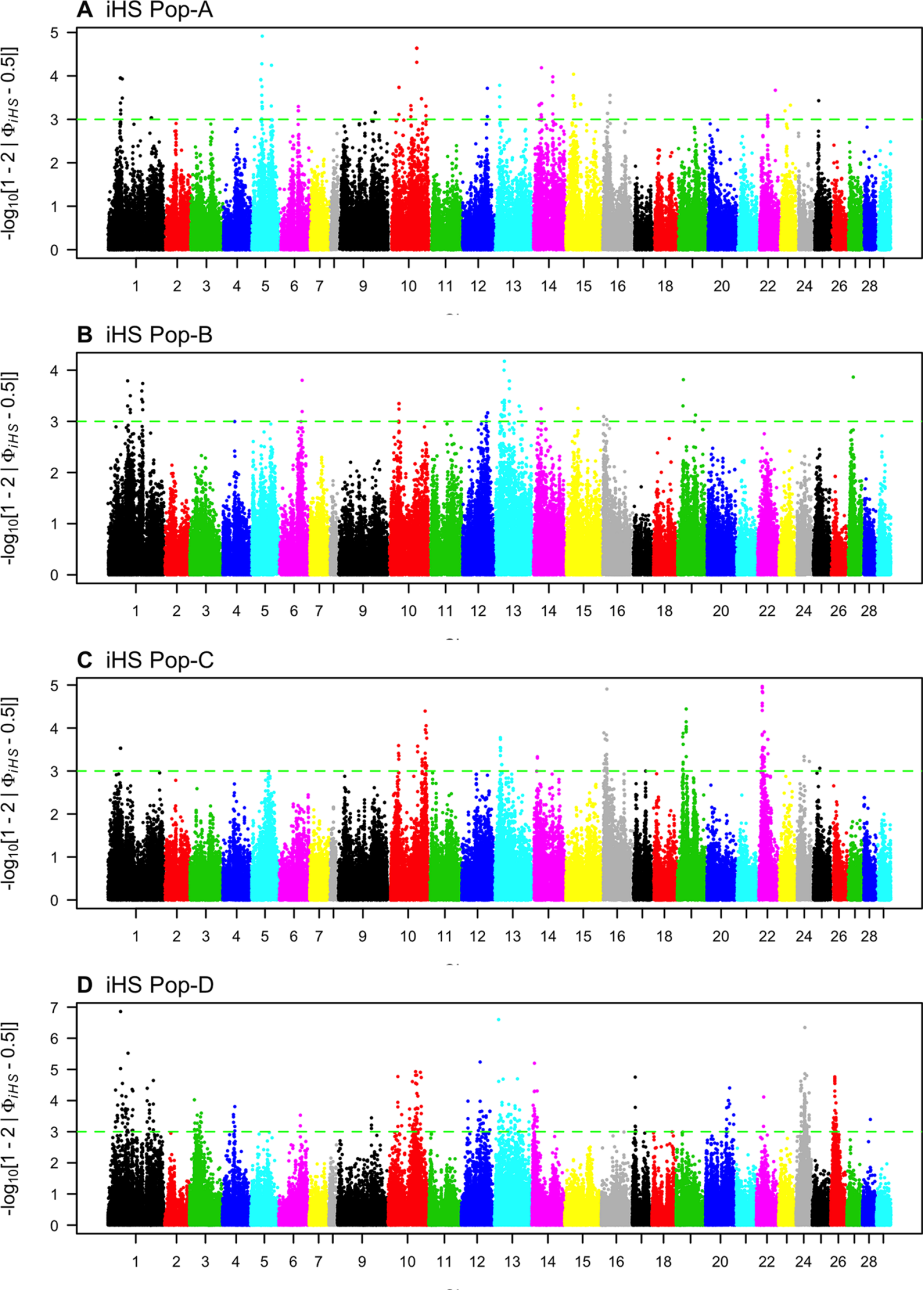
Genome-wide distribution of -log_10_(*P* value) of standardized integrated haplotype score (iHS) across four Atlantic salmon populations: **(A)** Pop-A, **(B)** Pop-B, **(C)** Pop-C, and **(D)** Pop-D.

We looked for evidence of selection by comparing the decay of association between alleles from the major versus minor allele at core SNPs using iHS. We found 115, 63, 142, and 467 core SNPs with significant iHS statistics (*p* ≤ 0.001) for Pop-A, -B, -C, and -D respectively (**Figure 4**, **Table 2**). We find 27, 12, 23, and 83 regions in these respective populations with at least two significant SNPs that are ≤ 500 kb apart, which we classify as putatively, selected regions.

**Table 2:** Ten genome regions spanning the strongest detected selection signatures by iHS in each population.

POP	CHR	START	END	-log(*p*)	|iHS|	N SNPs	SIZE (kb)
Pop-A	1	35662318	35684677	3.9515	3.8634	4	22.4
	1	40728165	40728699	3.9306	3.8516	2	0.5
	5	25918328	25932901	3.9169	3.8439	2	14.6
	5	28372137	29065939	4.2779	4.0432	5	693.8
	5	29574408	29842752	4.9158	4.3751	4	268.3
	5	55278111	55732536	4.2454	4.0256	2	454.4
	10	79382450	79401333	4.6374	4.2331	3	18.9
	14	24674586	25715785	4.188	3.9944	6	1041.2
	14	56736246	57120611	3.98	3.8794	4	384.4
	15	22773166	23073140	4.0388	3.9122	6	300
Pop-B	1	55995699	56003301	3.7914	3.7725	2	7.6
	1	63381907	63519535	3.5006	3.602	3	137.6
	1	95895287	95964374	3.5929	3.6568	2	69.1
	1	98490168	98568189	3.7394	3.7425	3	78
	6	65448844	65496448	3.8025	3.7788	2	47.6
	10	29948442	30759289	3.347	3.509	4	810.8
	12	71810541	71833088	3.1679	3.3978	2	22.5
	13	22110373	22139660	3.371	3.5237	3	29.3
	13	27127510	28267153	4.1737	3.9866	10	1139.6
	13	41965178	42139618	3.8649	3.8144	15	174.4
Pop-C	10	104686655	105233083	4.3935	4.1051	10	546.4
	10	107544485	107633657	4.0535	3.9204	4	89.2
	16	6030690	6249119	3.8867	3.8268	3	218.4
	16	12985501	13367987	3.8395	3.7999	4	382.5
	16	14071951	14921512	4.9062	4.3703	7	849.6
	19	16762570	17099010	3.7932	3.7735	3	336.4
	19	17814180	18048311	3.877	3.8213	4	234.1
	19	26510295	26774029	4.4423	4.131	7	263.7
	22	16108465	17678398	4.9676	4.401	23	1569.9
	22	21553029	22158359	3.9096	3.8398	6	605.3
Pop-D	1	36254384	36351335	6.8581	5.267	2	97
	1	57451430	57811304	5.5208	4.6699	2	359.9
	10	80280055	81084212	4.9294	4.3819	6	804.2
	10	83621134	84353833	4.8176	4.3255	9	732.7
	10	93572235	93962188	4.9072	4.3708	3	390
	12	53402445	54250457	5.2386	4.5345	6	848
	13	14998846	15196522	6.6012	5.1573	5	197.7
	14	8208052	9149527	5.1987	4.5151	10	941.5
	24	24577538	26845034	6.3462	5.0462	54	2267.5

Candidate regions for Pop-A were on Ssa01, Ssa05, and Ssa22. The candidate regions having SNPs with the most significant iHS scores were on Ssa05, Ssa10, and Ssa14, which contained the genes *igfbpl1* and *mipol1*.

Pop-B had 12 regions with an average length of ∼ 250 kb putatively under selection distributed among five chromosomes. The highest iHS score was for a region found on Ssa13 [-log(*p* value) = 4.17] containing 26 genes including the *soga1* gene. Pop-C had 23 candidate regions that were on average ∼370 kb long, and which spanned a total of 165 genes. The 1,570-kb-long region with one of the most significant iHS score was on Ssa22, and spanned the genes *kcnkf*, *sc61a*, and *mstn1*. Pop-D had the most significant number of SNPs (467) and had 83 putatively selected genomic regions under our criteria. Most of these regions were located on Ssa01, Ssa10, Ssa13, and Ssa26 and spanned genes, such as *haus2*, *itfg*1, and *phkb*. Details of the total regions and genes can be found in **Supplementary Tables S2** and **S5**, respectively.

### Candidate Regions Under Selection—*XP-EHH*

**Figure 5:**
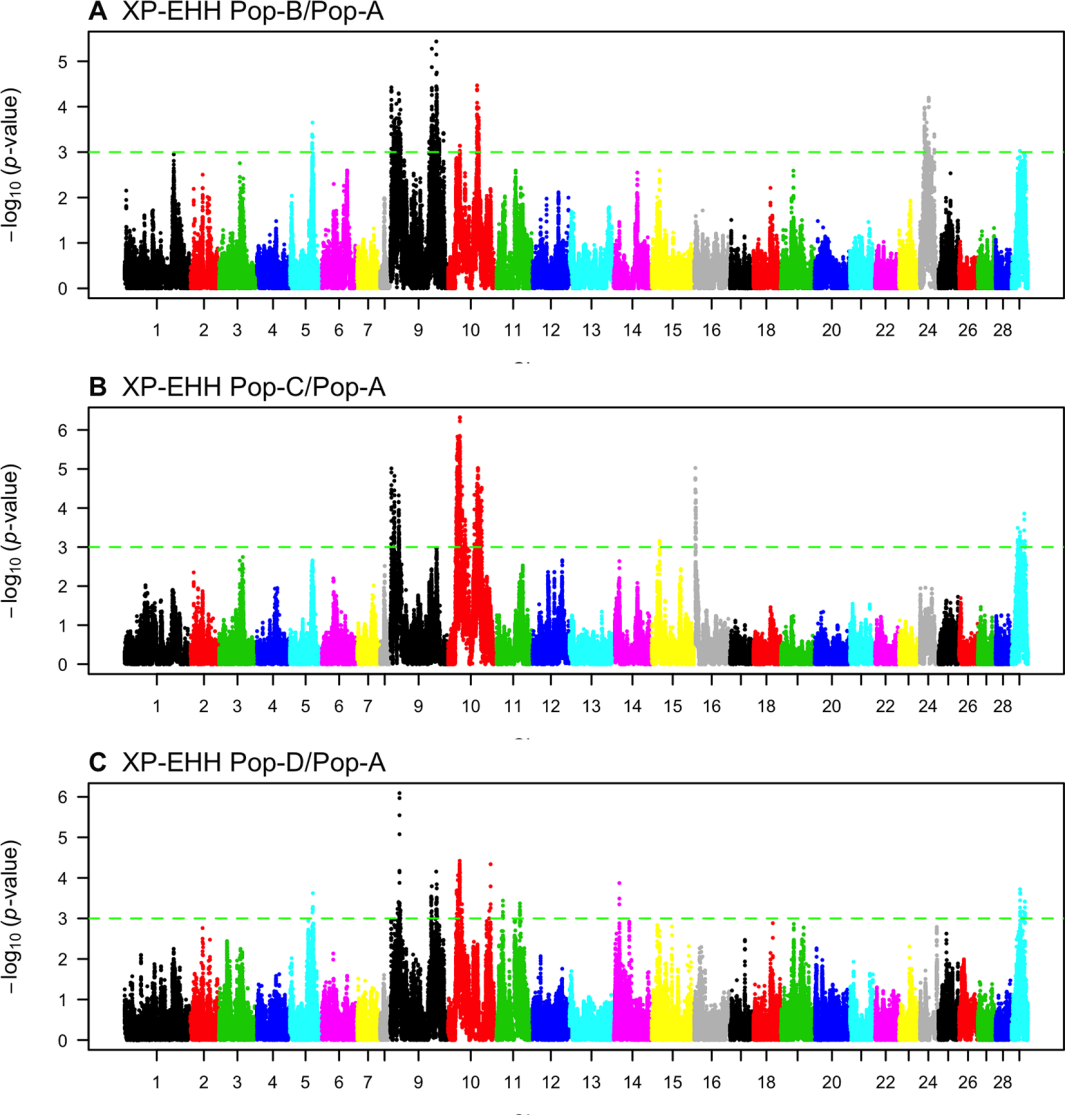
Genome-wide distribution of -log_10_(*P* value) of standardized cross-population extended haplotype homozygosity (XP-EHH) scores across three pairwise Atlantic salmon populations: **(A)** Pop-B/Pop-A, **(B)** Pop-C/Pop-A, and **(C)** Pop-D/Pop-A.

We compared the decay of LD from a core SNP as measured by EHH between the Norwegian source population and the three derived Chilean populations (Pop-B/Pop-A, Pop-C/Pop-A, Pop-D/Pop-A) to detect regions having unusually high EHH and overrepresented haplotypes consistent with selection. In total, we detected 482 (Pop-B/Pop-A), 800 (Pop-C/Pop-A), and 207 (Pop-D/Pop-A) XP-EHH outlier SNPs indicative of selection (**Figure 5**, **Table 3**). The sign of the XP-EHH score indicates which population selection is acting on. Here, negative scores suggest selection in Pop-A. Most significant SNPs, which we considered as those with XP-EHH score *p* ≤ 0.001, had negative scores, suggestive of selection in the Irish source population. The Pop-C/Pop-A and Pop-D/Pop-A comparisons yielded 38 and 3 significant SNPs with positive scores respectively, suggesting that the C and D populations underwent selection after their introduction into Chile. The significant, positive scores suggesting selection in Pop-C were found on Ssa16 within two regions spanning a total of 664.2 kb and which harbored 17 genes. The significant SNPs pointing to selection in Pop-D were located on Ssa14 in an 18.4-kb region, which contained the gene *agla*. XP-EHH did not detect selection signatures in Pop-B, as all significant scores for the Pop-B/Pop-A pair were negative. We classified potential genomic regions under selection as those containing two or more significant, adjacent SNPs less than 500 kb apart. After merging overlapping regions, we identified 34, 28, and 23 candidate regions from the Pop-B/Pop-A, Pop-C/Pop-A, and Pop-D/Pop-A comparisons respectively, which were all suggestive of selection in Pop-A. The average lengths of the candidate regions are approximately 338 kb for Pop-B/Pop-A, 546.5 kb for Pop-C/Pop-A, and 139 kb for Pop-D/Pop-A. Together, these regions span a total of 667 genes. Details of the total regions and genes detected by XP-EHH can be found in **Supplementary Table S3** and **S6**, respectively.

**Table 3:** Genome regions spanning the strongest detected selection signatures by XP-EHH in populations A, C and D.

POP	CHR	START	END	-log(*P*)	XP-EHH	N SNPs	Size (kB)
Pop-A	10	28741972	31140475	6.324	-5.0365	153	2398.5
	9	23728364	24144245	6.0912	-4.9328	15	415.9
	10	24160722	26099914	5.8281	-4.8132	127	1939.2
	9	113910288	114187655	5.4365	-4.6298	30	277.4
	10	21739331	23180890	5.3423	-4.5847	54	1441.6
	9	101786257	103293781	5.2766	-4.553	56	1507.5
	10	73472292	74738689	5.021	-4.4276	73	1266.4
	9	3674860	4026195	5.0148	-4.4245	14	351.3
	9	11161334	11559014	4.823	-4.3282	19	397.7
	9	114997862	115904242	4.7511	-4.2916	44	906.4
Pop-C	16	3564058	3808523	5.0253	3.2964	13	244.5
	16	4345514	4765204	4.4036	3.2915	25	419.7
Pop-D	14	14636389	14654809	3.872	3.5093	3	18.4

### Candidate Regions Under Selection—*BayeScan*

**Figure 6:**
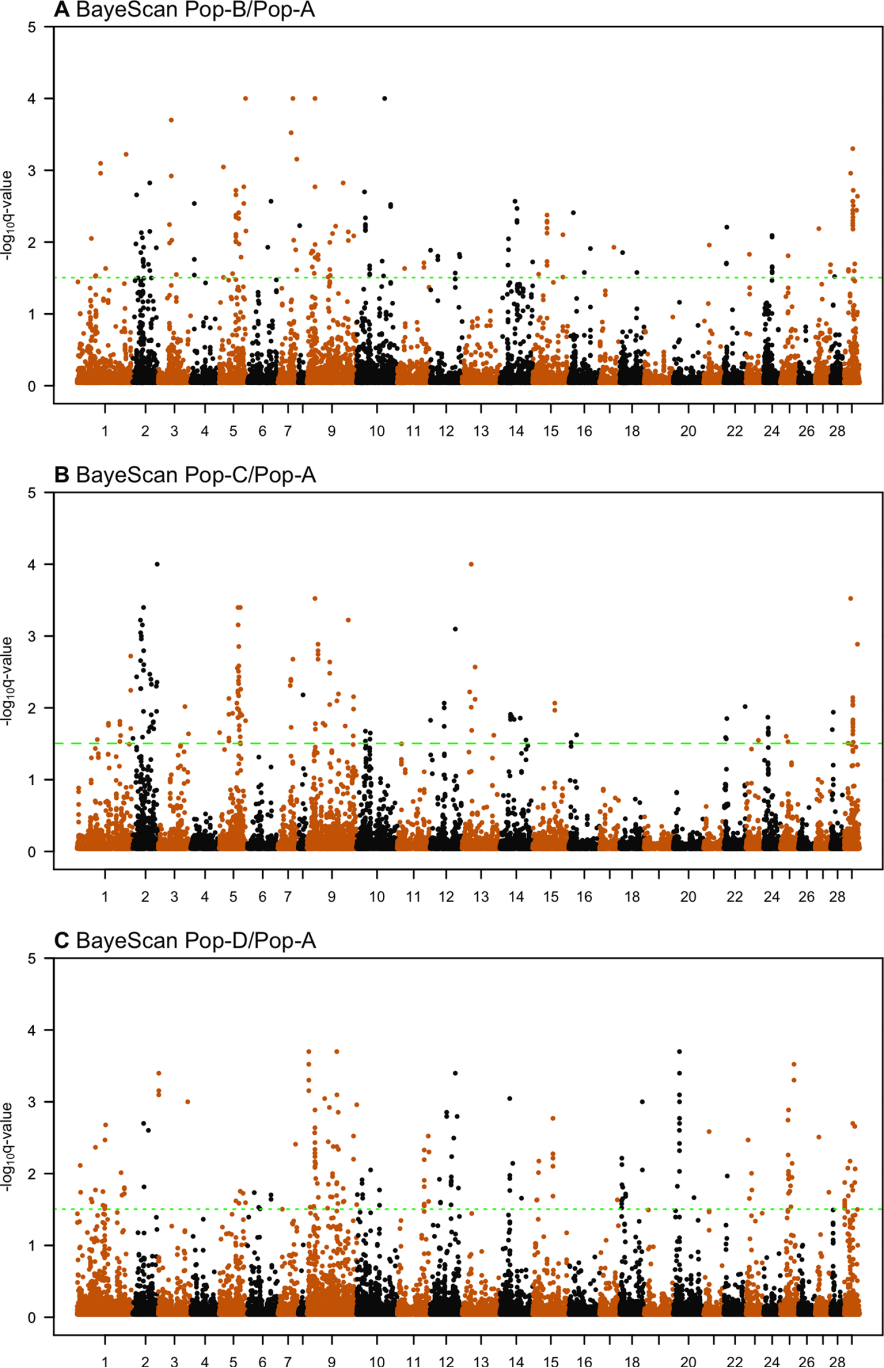
Genome-wide distribution of -log10(*q* value) in BayeScan analysis across three pairwise Atlantic salmon populations: **(A)** Pop-B/Pop-A, **(B)** Pop-C/Pop-A, and **(C)** Pop-D/Pop-A.

We used the Bayesian approach for estimating the posterior odds of selection acting at particular loci based on pairwise divergence between ancestral and derived populations implemented in BayeScan. By applying the BayeScan method to Pop-B/Pop-A, Pop-C/Pop-A, and Pop-D/Pop-A population pairs we, respectively, found 167, 155, and 193 SNPs with posterior odds ratios above 32, which was our threshold for showing significant evidence of selection (**Figure 6**, **Table 4**). F_ST_-based methods do not directly indicate in which population selection is acting; therefore, we describe our findings in terms of the population pairs. Since we expect regions that are truly under selection to have clusters of highly diverged SNPs in LD, we considered only regions containing at least two significant SNPs that were less than 500 kb adjacent to each other as being strong selection candidates. Under this criterion 104, 98, and 121 SNPs with posterior odds ratios of selection above 32 remain of interest for the Pop-B/Pop-A, Pop-C/Pop-A, and Pop-D/Pop-A comparisons, respectively. Clusters of SNPs identified as being in or adjacent to putatively selected regions from the Pop-B/Pop-A comparison represent 31 regions that are, on average, ∼96.8 kb long and which harbored 58 genes. The Pop-C/Pop-A comparison showed 98 highly diverged regions among 29 regions that were, on average, ∼220.7 kb long and which spanned 200 genes. Finally, the Pop-D/Pop-A comparison revealed 28 candidate regions that were, on average, ∼153.6 kb long and contained 130 genes. Only two SNPs among these candidate regions showed evidence of selection among the three population pairs, which were located on Ssa29 in association with the *kmt2ca* gene. Twenty SNPs suggestive of selection were shared between Pop-B/Pop-A and Pop-C/Pop-A and were associated to regions that harbor 12 genes *rabac1, znf1030, tpi1b, si:ch211-206a7.2, znf1041, lpcat3, atp1a3b, zgc:158654*, and *myh10* on Ssa02, *znf385d* on Ssa05, *agbl4* on Ssa10, and CR388166.1, and *kmt2ca* on Ssa29. Four candidate SNPs were common to Pop-C/Pop-A and Pop-D/Pop-A and two between Pop-B/Pop-A and Pop-D/Pop-A, which correspond to the *kmt2ca* gene shared among three population pairs. Details of the total regions and genes detected by BayeScan can be found in **Supplementary Tables S4** and **S7**, respectively.

**Table 4:** Ten genome regions spanning the strongest detected selection signatures by BayeScan method in each population pair.

POP	CHR	START	END	-log(*q* value)	N SNPs	SIZE (kb)
Pop-B/Pop-A	1	66642439	66648870	3.097	2	6.4
	2	48416445	48567681	2.824	3	151.2
	3	37023703	37052183	3.699	2	28.5
	5	11553116	11556394	3.046	2	3.3
	5	47250892	47494177	2.721	8	243.3
	5	69864532	69865664	2.770	2	1.1
	7	53824902	53839042	3.155	2	14.1
	9	22192894	22527645	4.000	4	334.8
	29	23820153	24379023	3.301	10	558.9
	29	25107616	25137079	2.721	6	29.5
Pop-C/Pop-A	2	21743330	22285719	3.222	4	542.4
	2	24203593	24203644	3.000	2	0.1
	2	27316859	27731651	3.155	2	414.8
	2	30394158	31352454	3.398	5	958.3
	2	69206072	69622942	4.000	2	416.9
	5	52915411	53613743	3.398	5	698.3
	5	59616884	59678559	3.398	4	61.7
	9	30961027	30994613	2.886	4	33.6
	13	25691366	25715347	4.000	2	24.0
	29	23852604	24289616	2.886	12	437.0
Pop-D/Pop-A	3	1316421	1317893	3.398	3	1.5
	9	4536926	4590569	3.699	4	53.6
	9	22080850	22146356	2.886	9	65.5
	9	84229138	85051608	3.699	6	822.5
	9	141700047	141700106	2.959	2	0.1
	14	28094905	28343120	3.046	4	248.2
	18	64503159	64648197	3.000	2	145.0
	20	17158525	17477234	3.699	10	318.7
	25	22716335	22760611	2.886	6	44.3
	25	38351034	38355710	3.523	2	4.7

### Gene Ontology for Candidate Genes Under Selection

To further explore the functions of the candidate genes spanned by regions showing evidence of selection from the iHS, XP-EHH, and BayeScan analyses, we annotated the candidate genes using the DAVID browser (https://david-d.ncifcrf.gov). The candidate genes were enriched in 37 gene ontology (GO) terms overall, most of them population specific (**Table 5**). Four GO categories were common between Pop-A and Pop-B (single-multicellular organism process, single-organism developmental process, regulation of metabolic process, and anatomical structure development) and one between Pop-C and Pop-D (animal organ development). The remaining GO categories were unique to each population.

**Table 5:** Biological processes enriched in genes detected by iHS and XP-EHH in each Atlantic salmon population.

Population	Biological Process	GO Term	%	*p*	Benjamini
Pop-A	Cellular metabolic process	GO:0044237	36.8	3.0E-4	3.7E-2
	Organic substance metabolic process	GO:0071704	38.7	9.4E-4	5.6E-2
	Primary metabolic process	GO:0044238	37.1	1.2E-3	4.8E-2
	Catabolic process	GO:0009056	5.7	2.3E-2	5.1E-1
	Single-multicellular organism process	GO:0044707	19.1	4.7E-2	7.0E-1
	Developmental induction	GO:0031128	0.4	5.9E-2	7.1E-1
	Single-organism developmental process	GO:0044767	19.2	6.7E-2	7.1E-1
	Regulation of metabolic process	GO:0019222	14.0	7.6E-2	7.0E-1
	Anatomical structure development	GO:0048856	19.1	9.7E-2	7.5E-1
Pop-B	Regulation of signaling	GO:0023051	14.5	8.8E-3	4.7E-1
	Regulation of cellular process	GO:0050794	45.2	1.4E-2	4.0E-1
	Regulation of metabolic process	GO:0019222	22.6	3.8E-2	6.0E-1
	Anatomical structure morphogenesis	GO:0009653	17.7	4.8E-2	5.9E-1
	Regulation of response to stimulus	GO:0048583	12.9	5.0E-2	5.2E-1
	Cellular component organization	GO:0016043	24.2	5.1E-2	4.7E-1
	Single-organism developmental process	GO:0044767	27.4	6.2E-2	4.8E-1
	Anatomical structure development	GO:0048856	27.4	6.6E-2	4.6E-1
	Single-multicellular organism process	GO:0044707	25.8	9.8E-2	5.6E-1
	Methylation	GO:0032259	4.8	9.9E-2	5.3E-1
Pop-C	Heart development	GO:0007507	5.3	2.4E-2	1.0E0
	Regulation of cell communication	GO:0010646	8.8	2.7E-2	9.7E-1
	Regulation of signal transduction	GO:0009966	8.2	2.8E-2	9.2E-1
	Animal organ development	GO:0048513	14.7	3.0E-2	8.8E-1
	Organ morphogenesis	GO:0009887	6.5	3.3E-2	8.4E-1
	Digestive tract development	GO:0048565	2.4	3.7E-2	8.2E-1
	Muscle system process	GO:0003012	2.4	4.9E-2	8.6E-1
	Tissue development	GO:0009888	9.4	6.3E-2	8.9E-1
	Cellular developmental process	GO:0048869	13.5	6.5E-2	8.7E-1
	Phosphorus metabolic process	GO:0006793	12.4	8.1E-2	9.0E-1
	System development	GO:0048731	17.6	9.7E-2	9.2E-1
Pop-D	Pancreas development	GO:0031016	1.7	5.1E-3	9.0E-1
	Cellular lipid metabolic process	GO:0044255	4.0	6.3E-3	7.6E-1
	Regulation of blood pressure	GO:0008217	1.0	1.1E-2	8.1E-1
	Lipid metabolic process	GO:0006629	4.7	1.6E-2	8.3E-1
	Gland development	GO:0048732	2.2	1.6E-2	7.7E-1
	Forebrain development	GO:0030900	1.5	2.6E-2	8.6E-1
	Small molecule metabolic process	GO:0044281	6.2	4.4E-2	9.5E-1
	Atrioventricular canal development	GO:0036302	0.5	5.0E-2	9.5E-1
	Organic acid metabolic process	GO:0006082	3.5	5.1E-2	9.3E-1
	Embryonic organ development	GO:0048568	3.5	7.0E-2	9.6E-1
	Animal organ development	GO:0048513	11.4	8.6E-2	9.7E-1
	Single-organism biosynthetic process	GO:0044711	4.2	9.2E-2	9.7E-1

## Discussion

In this study, we used three complementary tests to detect selection signatures within and between four Atlantic salmon populations with Norwegian origin. We used the iHS test to scan for selection signatures within populations and XP-EHH and BayeScan to find evidence of selection in terms of divergence of the Chilean populations to their ancestral Irish population. We detected several genomic regions under putative selection across all of the populations evaluated, which provides insight into the genes contributing to traits of importance to Atlantic salmon farming. It is important to mention that these findings should be interpreted with caution since other evolutionary and demographic process, such as bottlenecks and differences in the amount of genetic drift resulting from different effective populations sizes, can produce patterns of genetic diversity that mimic selection leading to the finding of possible false positives as well. However, the selection detection methods we used have all been shown to be robust to these confounding effects.

### Structure and Diversity

To examine genetic population structure and relationships among the major groups of salmon, we conducted an ADMIXTURE analyses based on high-quality SNP data. This analysis revealed that 12 ancestral lineages contribute to the modern gene pool represented by the four farmed populations, which was expected considering the admixed origin of these populations (Verspoor et al., 2007). The four populations used in this study are derived from the Mowi strain, which was created using samples from several rivers along the west coast of Norway (Norris et al., 1999). The population with the lowest level of admixture was Pop-A, which was also the population with the lowest genetic diversity, a condition that could reflect a better culture management, as well as intense artificial selection that erodes genetic variation through mating related individuals (Gjedrem, 2005). Pop-B and Pop-C which were introduced into the same region in Chile have very similar amounts of heterozygosity and similar degrees of admixture though the dominant lineages are different, which was expected due to the similar breeding practices and environmental conditions to which they have been subjected. Pop-D, however, showed the highest level of heterozygosity and a more complex pattern of admixture, whereby a single ancestral lineage is highly represented within individuals but with many ancestral lineages present among individuals. This pattern may, in part, reflect lower artificial selection pressure. Recent genetic introgression cannot be ruled out for Pop-D given the potential for crossing with different strains for management reasons. LD analysis revealed that overall LD decays more rapidly in Pop-B and Pop-C over short physical distances and is lower than Pop-A and Pop-D. The pattern of LD in Pop-A is consistent with its lower heterozygosity level. However, similar pattern was observed in Pop-D, likely due to higher level of admixture in this population, where several ancestral lineages can be observed. Chromosomal LD decay followed similar patterns, but in Pop-A, LD decay was noticeably higher in chromosomes 2, 9, 11, 19, and 29, which is agreed with a greater number of regions detected under selection in those chromosomes. Conversely, in chromosome 26, Pop-D showed the highest value of LD (*r*^2^ = 0.12), probably related to a larger region under selection detected in this population. The results presented here also reinforce the notion that exposure to different management and environmental conditions over just a few generations (at least four in this particular case) is sufficient to generate large changes in the genetic structure of farmed Atlantic salmon populations with the same genetic origin.

### Selection Signatures

**Figure 7:**
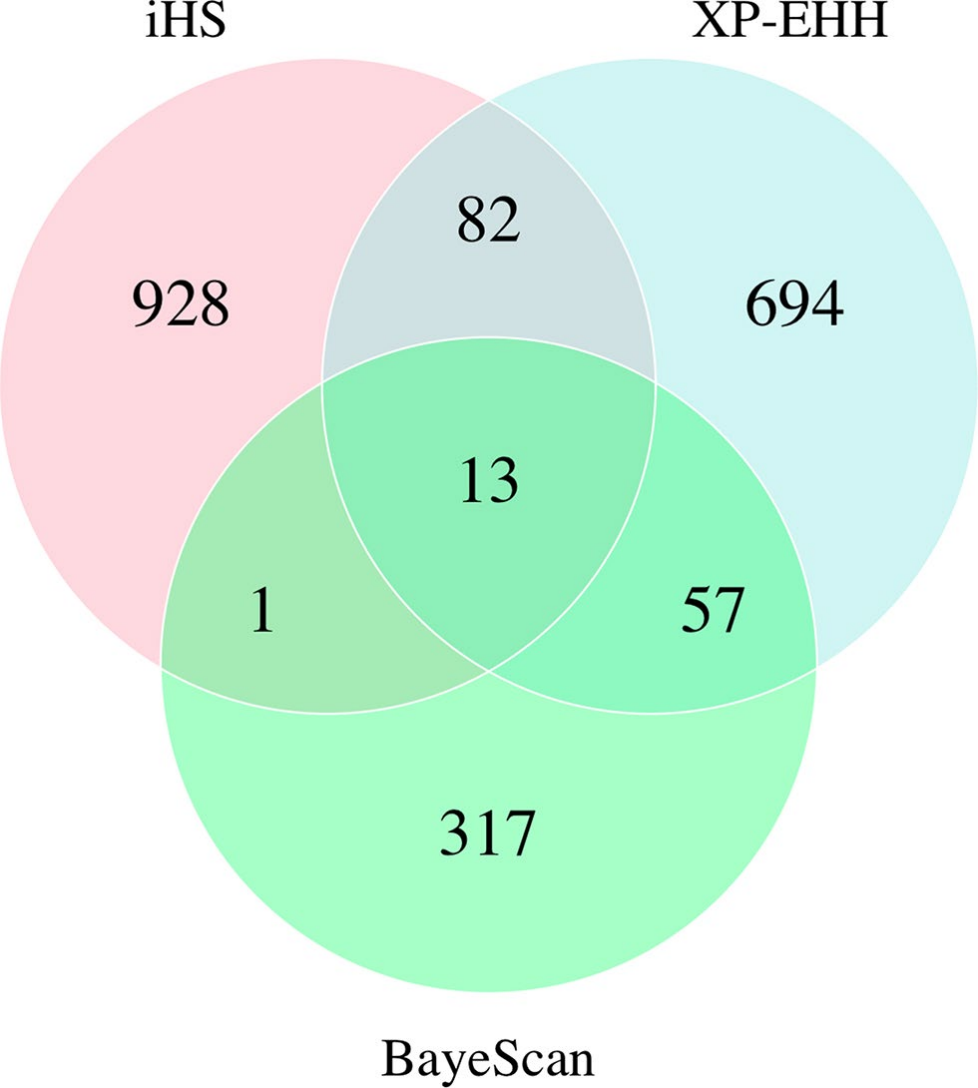
Venn diagram showing shared genes identified among three independent tests in the four populations of Atlantic salmon.

Pop-D had regions showing the strongest evidence for selection as well as the most candidate regions according to the iHS test. Although the iHS test has a lower power to detect selection under nearly complete sweeps (Sabeti et al., 2007; Simianer et al., 2010), it has greater power when selected alleles are at intermediate frequencies. Pop-D has experienced weaker artificial selection pressure than the other populations used in this study (Jean Paul Lhorente, personal communication), and so the higher number of putatively selected regions identified in this population by iHS may reflect more sweeps at intermediate frequencies because they are taking relatively longer to complete under weaker selection. In addition, this population is located in the Magallanes Region in Chile, which exposes salmon to more extreme environmental conditions than in the Los Lagos region where Pop-B and Pop-C were introduced. Therefore, the selection imposed by the natural environmental may also contribute to a relatively high number of selected regions in Pop-D. In contrast to iHS, XP-EHH is powerful at detecting complete or nearly complete selective sweeps (Sabeti et al., 2007). According to the XP-EHH method, Pop-A shows the greatest number of regions under selection across the genome, which is consistent with XP-EHH having greater power to identify selection in regions that experienced older selection events (Sabeti et al., 2007; Klimentidis et al., 2011) than iHS since Pop-A is the oldest population in the present study while also being subjected to more intense artificial selection. We identified several putative directional selection targets using BayeScan, but given the nature of F_ST_-based methods we are unable to directly identify which population in a pairwise comparison is experiencing selection from the posterior odds alone. Low overlap in selected regions identified with haplotype-based and single-SNP F_ST_-based approaches have been reported in other studies in Atlantic salmon (Mäkinen et al., 2014; López et al., 2018) and other species (Bahbahani et al., 2015). However, we did find some degree of overlap among genes detected by both haplotype methods and the F_ST_ method as shown in **Figure 7** and **Table 6**.

**Table 6:** Genes detected by at least two selection signatures methods. Genes are indicated in the left column and in the right column their corresponding methods.

GENES	METHODS
CRISP3, NOTCHL, GPSM1B, SI : ZFOS-367G9.1, PHF1, FQ976914.1, TAP1, PBX2, DNASE2, RGL2, PLCL2, SYNGAP1B, BRD2A	iHS; XP-EHH; BayeScan
CRISP3, SI : ZFOS-367G9.1, NOTCHL, GPSM1B, PHF1, FQ976914.1, TAP1, PBX2, DNASE2, RGL2, PLCL2, SYNGAP1B, BRD2A, DOCK10, CRK, LRRC75A, SI : CH211-232I5.3, BLOC1S2, SI : DKEYP-51F12.3, CEP120, CABZ01077978.1, SI : CH211-232I5.1, PRKAA1, PLPP3, BX546500.1, DHCR24, USP24, DAB1A, PRDM5, ANAPC4, SLC10A4, FRYL, PALLD, SLAIN2, MOGAT3B, C1QTNF7, FTR14, LRRC66, SGCB, RASL11B, NDNF, ZBTB34, CPEB2, CC2D2A, FBXL5, NEK1, SH3RF1, OCIAD2, DCUN1D4, USP46, OCIAD1, SCFD2, CDKN1BB, YARS2, PPARAB, BX537249.1, JPH3, KLHDC4, SLC7A5, HMCN2, CDH13, RANBP10, NUTF2, EDC4, NRN1LA, MBTPS1, SLC38A8, PNP6, CALB2A, PSKH1, NECAB2, SCAPER, PSTPIP1A, THBS4A, SERINC5, TRAFD1, SMTNB, UBE2G1B, ANAPC7, ADORA2AA, GUCD1, TAS2R200.1, GSTT1A, DERL3, SMARCB1A, ATP2A2A, BCR, SPECC1LA, SI : CH211-191O15.6, SNRPD3, P2RX7, MMP11A, RALGDS, IFT81, MPEG1.1	iHS; XP-EHH
UNC13B, CRISP3, SI : ZFOS-367G9.1, NOTCHL, GPSM1B, PHF1, FQ976914.1, TAP1, PBX2, DNASE2, RGL2, PLCL2, SYNGAP1B, BRD2A	iHS; BayeScan
NXPH1, ICA1, MIOS, COL28A1B, TAC1, SEPT7B, NEK10, NR1D2B, PHLPP1, RAB5AB, EFHB, CRISP3, SI : ZFOS-367G9.1, NOTCHL, GPSM1B, PHF1, GLCCI1, COL28A1A, RARGA, UBE2E2, ZNF385D, SATB1, FQ976914.1, TAP1, PBX2, DNASE2, RGL2, PLCL2, SYNGAP1B, BRD2A, CTSS2.1, STARD13A, VASH1, OLFM4, RPS6KL1, AREL1, FCF1, ANGEL1, DLST, ESRRB, GPATCH2, TGFB3, PROX2, TMEM179, ARHGEF18B, CABZ01071407.1, ATXN3, SERPINA10, FOXP1A, SI : DKEY-206P8.1, DDX24, SI : CH1073-416D2.4, PRIMA1, UBR7, ITPK1B, HSPA4L, MRPL35, SI : DKEY-21A6.5, CABZ01052815.1, CABZ01066926.1, CHMP3, REEP1, BTBD7, PLK4, MYO1CB, AGBL4, MYL2B, PPP1CC, MTMR3, CUX2B	XP-EHH; BayeScan

### Biological Function of Candidate Selected Regions

Geographical adaptation and selection in farmed Atlantic salmon has resulted in considerable differences between wild and farmed strains (Glover et al., 2009). Genomic regions detected in this study strongly suggest selection on traits that could be associated with either natural or artificial selection, as they relate to the immune system, growth, and behavior, which are all often altered through domestication. Growth has been the main trait focused on by the breeding programs represented by our focal salmon populations. In agreement with this, we found several genes showing evidence of selection that could be potentially influencing growth such as *chp2* and *ccser1*, which were associated with body weight in a previous genome-wide association study (GWAS) on Atlantic salmon (Yoshida et al., 2017). We detected the *kind1* gene that is also associated with growth traits in juvenile, farmed Atlantic salmon (Tsai et al., 2015). It has also been shown that insulin growth factors (IGFs), IGF receptors, and IGF binding proteins, play an important role in regulating growth in several teleost fish species (Duan, 1997). We detected the IGF 1-receptor (*igf1r*), IGF binding protein 6 paralog A2 (*igfbp-6a2*), and IGF binding protein-related protein 1 precursor (*igfbprp1*) as being under selection. We hypothesize that these genes are all contributing to weight variation in farmed salmon. The GO analyses for our candidate genes also showed enrichment for categories related to metabolic and developmental processes, which could certainly affect growth.

Genes functioning in host–pathogen interactions may be targets of natural selection more often than genes from other functional categories (Schlenke and Begun, 2003). The populations used in this study have not been artificially selected for disease resistance; however, we suspect that the culture environment has imposed natural selection on regions implicated in immune system function. We found evidence of selection in seven genes (*kcnb2*, *rlf*, *synrg*, *snx14*, *fbxl5*, *e2f4*, *blm*) that were previously shown to be affected by parasite-driven selection (Zueva et al., 2014). We also identified three genes potentially under selection *(kcnq1*, *lrp5*, and *sh3rf1)* that have were associated with disease resistance in the face of a bacterial disease (*Piscirickettsia salmonis*) in Coho salmon (Barría et al., 2018) and *mettl12* which is associated with immune response to parasites in three-spined stickleback (Huang et al., 2016).

Behavioral traits are among the first traits affected by animal domestication (Kohane and Parsons, 1988), and it has been suggested that domestication may impact behavior even after only one generation (Huntingford, 2004). Among our candidate genes putatively under selection, we identified the endoplasmic reticulum protein 27 (*erp27*) gene, the differential expression of which has been associated to tameness in the red junglefowl (Bélteky et al., 2016). Also, among our candidates were genes, such as *gabrb1*, *scaper*, *clstn3*, and *pex5*, related to mental disorders in humans such alcoholism and schizophrenia (Glatt et al., 2005; Enoch, 2008; Pettem et al., 2013). We think that these genes may be influencing behavior in the salmon populations we studied, and that the artificial selection and domestication could be acting inadvertently on the traits affected by these genes like those that occur in other domestic animals (Clutton-Brock, 1999).

In salmon culture, early sexual maturation has undesired consequences, such as decreased growth and feed conversion efficiency (Good and Davidson, 2016). To avoid these negative effects, maturation is commonly delayed by exposing fish to continuous light, which affects the perception of seasonality and circannual rhythms (Taranger et al., 2010). We would expect then to find genes underlying traits related to maturation rate as showing signs of selection, which we apparently do. One putatively selected gene that we found that may affect maturation rate is *akap13*, which has been shown to play a role in ovarian development in human (Wu et al., 2015), as well as a gene in the AKAP (*akap11*) family, which was previously associated with age to maturity in Atlantic salmon (Barson et al., 2015).

Other interesting genes spanned by regions showing evidence for selection in this study are *hao1*, which is associated with chicken sexual ornaments (comb size), *myo3a*, which is involved in allowing dogs to sense local environmental stimuli (Wang et al., 2013), and *pgbd4*, which is considered a candidate gene involved in adaptation at the regional scale in Atlantic salmon (Bourret et al., 2013) and so could be functioning in adaptation to the aquaculture environment.

## Conclusions

To summarize, in this study we used three different but complementary statistical approaches, iHS, XP-EHH, and BayeScan to detect selection signatures in four farmed Atlantic salmon populations with the same geographical origin, but adapted to different environmental conditions. The methods used in this study were useful for detecting selection signals across populations and allowed us to find genes that could be related to growth, immune system function, and behavior in this species, characters that are commonly influenced by domestication. This study provides potential candidate genes for traits with both biological and economic importance for Atlantic salmon and establishes a strong platform for further studies seeking to better understand how particular genomic variants influence the evolution and cultivation of this species.

## Ethics Statement

The sampling protocol was previously approved by The Comité de Bioética Animal, Facultad de Ciencias Veterinarias y Pecuarias, Universidad de Chile (certificate 29-2014).

## Author Contributions

ML and JY conceived the research idea. ML drafted the manuscript and carried out the analyses. TL supervised the data analyses and contributed to discussion and writing. TL, AN, JL, RN, and JY reviewed the manuscript. All authors read and approved the final manuscript.

## Funding

This work has been conceived on the frame of the grant CORFO (11IEI-12843 and 12PIE17669), Government of Chile.

## Conflict of Interest

AN was employed by Marine Harvest, Kindrum, Fanad, C. Donegal, Ireland. JL was employed by company Benchmark Genetics Chile, Puerto Montt, Chile. The remaining authors declare that the research was conducted in the absence of any commercial or financial relationships that could be construed as a potential conflict of interest.
