# Supplementary material for: Multiple Selection Signatures in Farmed Atlantic Salmon Adapted to Different Environments Across Hemispheres
Source: Front Genet. 2019 Oct 1;10:901. doi: 10.3389/fgene.2019.00901 (PMC6786245; doi:10.3389/fgene.2019.00901)

Figure S1. Decay of linkage disequilibrium (LD) by chromosome for each population. Different color lines represent populations: Pop-A=Red, Pop-B=Green; Pop-C=Turquoise and Pop-D=Purple.

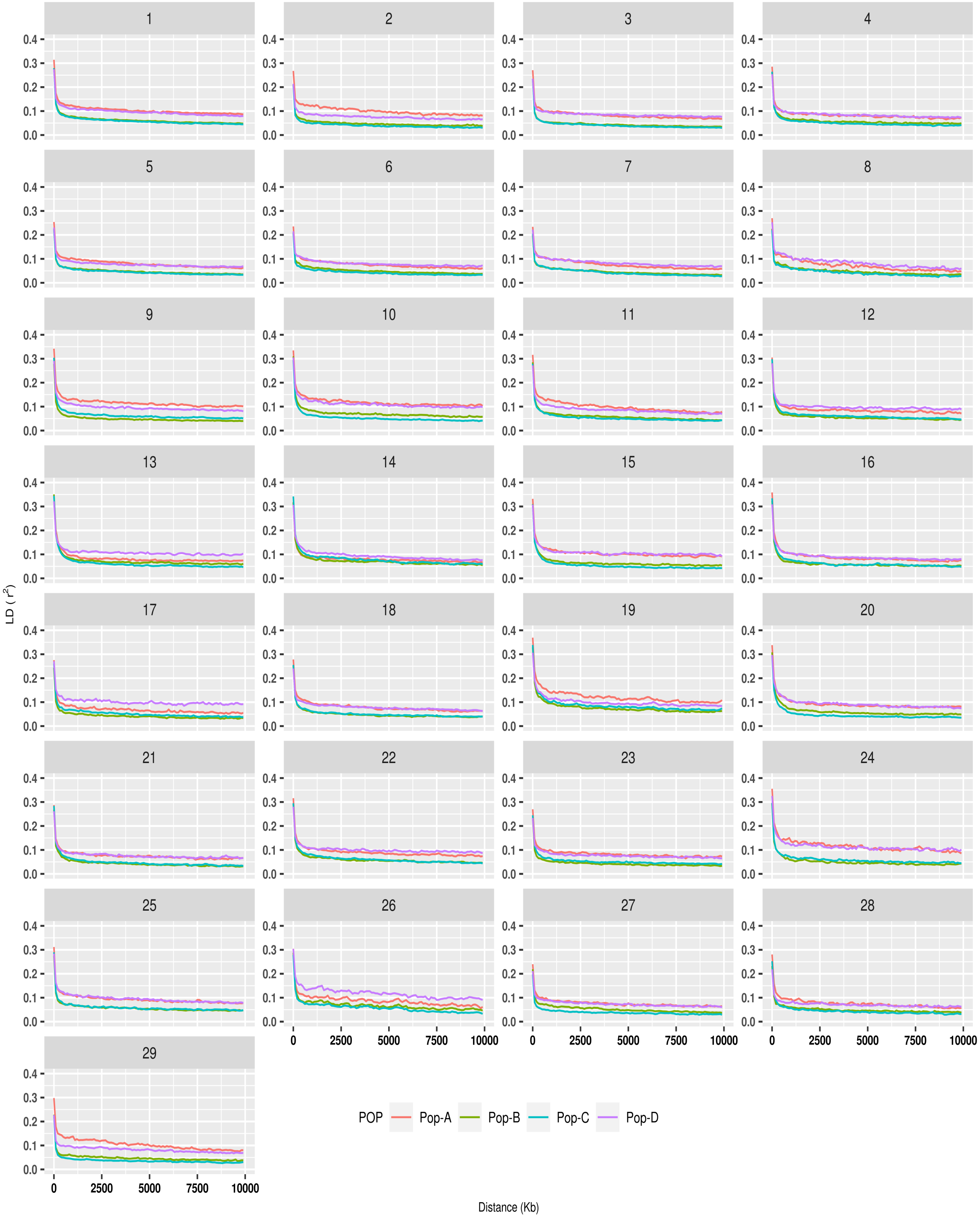

Supplement: Supplementary file 2 [file Image_1.pdf]
